# Antioxidant Capacity of Polar and Non-Polar Extracts of Four African Green Leafy Vegetables and Correlation with Polyphenol and Carotenoid Contents

**DOI:** 10.3390/antiox12091726

**Published:** 2023-09-06

**Authors:** Nelly Fioroni, Claire Mouquet-Rivier, Emmanuelle Meudec, Véronique Cheynier, Frédéric Boudard, Youna Hemery, Caroline Laurent-Babot

**Affiliations:** 1 UMR QualiSud, University of Montpellier, Avignon University, CIRAD, Institut Agro, IRD, University of La Réunion, 34090 Montpellier, France; claire.mouquet@ird.fr (C.M.-R.); frederic.boudard@umontpellier.fr (F.B.); youna.hemery@ird.fr (Y.H.); 2 SPO, INRAE, University of Montpellier, Institut Agro, 34060 Montpellier, France; emmanuelle.meudec@inrae.fr (E.M.); veronique.cheynier@inrae.fr (V.C.); 3 INRAE, PROBE Research Infrastructure, Polyphenol Analytical Facility, 34060 Montpellier, France

**Keywords:** bioactive compounds, flavonoids, β-carotene, lutein, *Manihot esculenta*, *Hibiscus sabdariffa*, *Corchorus olitorius*, *Amaranthus* spp., *Spinacia oleracea*

## Abstract

In sub-Saharan Africa, chronic malnutrition is often associated with intestinal inflammation and oxidative stress. African green leafy vegetables (GLVs), commonly consumed by these populations and rich in bioactive compounds, may improve the antioxidant status. The aim of this study was to measure the antioxidant capacity using complementary assays (DPPH, FRAP, ABTS, ORAC and NO scavenging) in polar and non-polar leaf extracts of four African GLVs, cassava (*Manihot esculenta*), roselle (*Hibiscus sabdariffa*), jute mallow (*Corchorus olitorius*), and amaranth (*Amaranthus* spp.), with spinach (*Spinacia oleracea*) chosen as a reference. Their antioxidant capacity was correlated with their total polyphenol (TPC), flavonoid (TFC), condensed tannin, lutein, and β-carotene contents. Identification of phenolic compounds by UHPLC-DAD-MS/MS revealed the presence of three main classes of compound: flavonols, flavones, and hydroxycinnamic acids. Cassava and roselle leaves presented significantly higher TPC and TFC than amaranth, jute mallow, and spinach. They also exhibited the highest antioxidant capacity, even higher than that of spinach, which is known for its important antioxidant effect. The antioxidant capacity was 2 to 18 times higher in polar than non-polar extracts, and was more strongly correlated with TPC and TFC (R > 0.8) than with β-carotene and lutein contents. These findings provide new data especially for cassava and roselle leaves, for which studies are scarce, suggesting an appreciable antioxidant capacity compared with other leafy vegetables.

## 1. Introduction

Malnutrition is a global public health issue. In 2020, stunting still affects 22% of <5-year-old children, particularly in sub-Saharan Africa (32%) [1,2]. Enteric infections and diarrheal episodes are among the main causes of chronic malnutrition [3]. These infections are often associated with environmental enteric dysfunction, characterized by acute intestinal inflammation potentially related to oxidative stress [4]. In these stressful conditions, the production of highly reactive pro-oxidant chemical species, such as free radicals (superoxide, hydroxyl radical, singlet oxygen, peroxyl), cannot be neutralized by the body’s antioxidant defenses [5,6]. This imbalance disrupts reduction–oxidation (redox) reactions, triggering oxidative stress, which can lead to tissue damage and loss of the intestinal barrier homeostasis [7]. This creates a condition of chronic inflammation that further damages the intestinal mucosa and exacerbates the problem [8,9].

The most appropriate strategy to prevent chronic oxidative stress is to improve the body’s antioxidant status by increasing the consumption of foods rich in bioactive compounds, such as fruits and vegetables with antioxidant and anti-inflammatory properties [10,11,12]. In sub-Saharan Africa, green leafy vegetables (GLVs) are widely consumed and have been identified as key foods due to their positive role in nutrition and dietary diversity. Traditionally, GLVs are cooked and eaten as a sauce with a starchy staple food [13,14]. African GLVs are rich in antioxidants, such as polyphenols (e.g., phenolic acids: hydroxybenzoic and hydroxycinnamic acids or flavonoids) [15,16,17,18] and carotenoids (e.g., lutein and β-carotene) [10,19], that give them a high antioxidant capacity [14]. Bioactive secondary metabolites found in vegetables can act through different mechanisms, such as free radical scavenging, mineral chelation, oxidative enzyme inhibition, and antioxidant enzyme cofactor induction [20,21]. Vitamin C and E and carotenoids and polyphenols are considered the main antioxidants provided by the diet. Many studies demonstrated that polyphenols have a higher antioxidant capacity than carotenoids and vitamins [22,23,24]. Polyphenols constitute the largest group of plant secondary metabolites among which flavonoids are the most prevalent and diverse subgroup [25,26]. Polyphenol antioxidant capacity is attributed to their ability to scavenge reactive oxygen species (ROS) due to their electron- or hydrogen-donating properties, to bind to transition metal ions as inert complexes, and to regenerate the potent chain-breaking antioxidant α-tocopherol [27]. Vitamin C can be defined as a free-radical scavenger. As an electron donor/acceptor, it is a potent water-soluble antioxidant in humans. Moreover, vitamin C can regenerate α-tocopherol, a form of vitamin E [28]. Vitamin E is a fat-soluble antioxidant that plays a crucial role in the protection against lipid peroxidation, peroxyl radical scavenging, and reductions in ROS and reactive nitrogen species (RNS) [20]. Carotenoids belong to a group of fat-soluble antioxidants, some of which are vitamin A precursors (e.g., β-carotene). Due to conjugated double bonds, they are quenchers of the highly reactive form of oxygen known as singlet oxygen and also radical scavengers, particularly to stop lipid peroxidation [29,30]. Based on their structure and solubility, these antioxidants are classified into two groups: (i) polar compounds, including polyphenols and vitamin C, and (ii) non-polar compounds, mostly carotenoids, vitamin E, and chlorophylls. In each class, the degree of solubility may be different. For example, among carotenoids, lutein is more water-soluble than β-carotene due to its structural features [22,31].

The antioxidant capacity is usually estimated using a combination of several assays because of the diversity of modes of action of antioxidant compounds. Depending on the chemical reactions involved, assays can be categorized into two types: (i) assays based on redox reactions involving a single electron transfer, such as the 2,2-azinobis (3-ethyl-benzothiazoline-6-sulfonic acid) (ABTS), 2,2-diphenyl-1-picrylhydrazyl (DPPH), ferric reducing/antioxidant power (FRAP) and nitric oxide (NO) scavenging assays; and (ii) assays based on hydrogen atom transfer, such as the oxygen radical absorption capacity (ORAC) assay [21,32,33].

Most studies on African GLVs focused on the micronutrient and antinutrient composition [34,35], whereas data on their contents in bioactive substances and their involvement in the prevention of oxidative stress-related diseases are lacking. Recent studies have evaluated the antioxidant capacity of GLV extracts (polar and/or non-polar) using different in vitro methods. However, some GLVs are more often studied, probably because they are the most popular and commonly consumed, such as amaranth and jute mallow leaves (Katerere et al., 2012, Adeosun et al., 2016). Data on other GLVs, such as roselle and cassava leaves, are scarce, although they are part of the diet in many African countries [36,37].

This study focused on four African GLVs that are commonly consumed by populations in sub-Saharan Africa: amaranth (*Amaranthus* spp.), jute mallow (*Corchorus olitorius*), cassava (*Manihot esculenta*), and roselle (*Hibiscus sabdariffa*). Their total polyphenol content (TPC), total flavonoid content (TFC), condensed tannin content (CTC), profile of phenolic compounds and carotenoid content were characterized. Then, the antioxidant capacity of polar and non-polar extracts of such GLVs was evaluated using five in vitro methods (DPPH, ABTS, FRAP, ORAC, and NO scavenging). The strength of the linear relationships between antioxidant capacity and bioactive compound (TPC, TFC, CTC, β-carotene, and lutein) content was evaluated with Pearson’s correlation coefficients. These findings provide new data, especially for cassava and roselle leaves for which studies are scarce.

## 2. Materials and Methods

### 2.1. Plant Materials 

Four frozen African leafy vegetables (one batch per species) were purchased from African grocery stores in Montpellier (France) in 2021: amaranth (*Amaranthus* spp.), jute mallow (*C. olitorius*), cassava (*M. esculenta*), and roselle (*H. sabdariffa*). For comparison, fresh spinach leaves (*S. oleracea* L.) were purchased in a supermarket in Montpellier (France) in 2021. Spinach was chosen as reference because it is a GLV commonly considered as a functional food due to its interesting nutritional composition. Spinach is rich in vitamins (K, A, B9, C), minerals (magnesium, potassium, iron), and bioactive compounds (polyphenols, especially flavonoid derivatives of patuletin or spinacetin, flavones, and carotenoids) with antioxidant, anti-inflammatory, and anti-cancer properties [38]. All leaves were freeze-dried, crushed, and stored in plastic pots at −20 °C.

### 2.2. Chemical Reagents 

Standards of gallic acid, quercetin, cyanidin chloride, caffeic acid, quercetin-3-*O*-glucoside and tryptophan were purchased from Sigma-Aldrich (Saint-Quentin Fallavier, France). Folin-Ciocalteu’s reagent (10%), aluminum chloride (AlCl_3_) (10%), potassium acetate (1 M), DPPH, Trolox (6-hydroxy-2,5,7,8-tetramethylchroman-2-carboxylic acid), ABTS (7 mM), potassium persulfate (2.45 mM), ferric chloride (FeCl_3_·6H_2_O), 2,4,6-tri(2-pyridyl)-s-triazine (TPTZ), methyl-tert-butyl-ether (MTBE),2,2’-Azobis(2-amidinopropane) dihydrochloride (AAPH), fluorescein, sodium nitroprusside (5 mM), sulphanilamide, n-(1-naphthyl) ethylenediamine dihydrochloride phosphate buffered saline (PBS) acetate buffer, phosphate buffer, and methanol were supplied by Sigma-Aldrich (Saint-Quentin Fallavier, France). Ethanol, hexane, acetone, formic acid, and sodium carbonate (75 g/L), were purchased from Carlo Erba Reagents (Val de Reuil, France); hydrochloric acid, butanol, acetonitrile, ethyl acetate, phosphoric acid, and ferric ammonium sulfate (2%) were from HoneyWell Riedel-de Haën (Seelze, Germany). All solvents were of the highest analytical grade.

### 2.3. Preparation of Leaf Extracts

#### 2.3.1. Solid-Phase Polar Extraction

A weighed amount of each powdered freeze-dried leafy vegetable (~0.4 g) was suspended in 10 mL of extraction solvent (water/ethanol, 20/80, *v*/*v*) and stirred at room temperature for 30 min. After filtration with Whatman no. 1 filter paper, extracts were stored at −20 °C until the determination of total polyphenol, flavonoid, and condensed tannin contents and the measurement of the hydrophilic antioxidant capacity. Three extractions were made for each leafy vegetable.

#### 2.3.2. Solid-Phase Non-Polar Extraction

A weighed amount of freeze-dried leafy vegetables (~0.5 g) was stirred with 10 mL of extraction solvent (ethanol/hexane 4/3, *v*/*v*). After addition of 10 mL of 10% NaCl (p/v), the solution was vortexed for 1 min and centrifuged (Heraeus Multifuge X1R Refrigerated Centrifuge, ThermoFisher Scientific, Osterode, Germany) at 8500× *g* for 5 min. The lower phase was removed and the pellet was extracted with 10 mL of extraction solvent and 10 mL of milliQ water. Both upper ethanol/hexane phases were collected and evaporated to dryness under nitrogen (Organomation 12 Position N-EVAP Nitrogen Evaporator, ThermoFisher Scientific, Berlin, MA, USA). The dried extract was dissolved in 2 mL of acetone or ethanol, according to the analysis. Non-polar extracts were stored at −20 °C until the determination of carotenoid content and lipophilic antioxidant capacity. Three extractions were made for each leafy vegetable.

### 2.4. Determination of Total Phenolic Content (TPC), Total Flavonoid Content (TFC), and Condensed Tannin Content (CTC) in Polar Extracts

The TPC of raw polar extracts was determined using the method reported by Singleton et al. (1999) [39]. Interfering water-soluble components (reducing sugars, ascorbic acid) also were determined following the protocol developed by Georgé et al. (2005) [40] to remove them from the raw extracts. These components were recovered by solid-phase extraction using an Oasis hydrophilic-lipophilic balance (HLB) cartridge from Waters. The recovered volume of the washing extract was carefully measured. Appropriate dilutions of the raw extract and washing extract (0.5 mL) were oxidized with 2.5 mL of milliQ water-diluted with Folin-Ciocalteu’s reagent (10%) at room temperature for 2 min and then neutralized with 2.0 mL of sodium carbonate (75 g/L). The reaction mixture was incubated at 30 °C for 30 min, and absorbance was measured at 760 nm using a Spectro UV–Vis Infinite 200 Pro NanoQuant (Tecan Group Ltd., Grödig, Austria) (also used for all subsequent methods that require an absorbance reading). The TPC was determined by subtracting the gallic acid equivalent of the washing extract from that of the raw extract, and was expressed as milligrams of gallic acid equivalent (GAE) per 100 g dry matter (DM).

The TFC of polar extracts was determined with the method of (Oboh et al., 2009) [24] adapted from (Dowd, 1959) [41]. Appropriate dilutions of each extract (0.5 mL) were mixed with 0.5 mL of methanol, 50 µL of 10% AlCl_3_, 50 µL of 1 M potassium acetate, and 1.4 mL milliQ water and incubated at room temperature for 30 min. Absorbance was measured at 415 nm. The TFC was calculated using quercetin as standard and expressed as milligram quercetin equivalent (QE) per 100 g DM.

The CTC was determined using the butanol-HCl assay as described by Porter et al. (1986) [42]. Briefly, 6 mL of butanol/HCl reagent (95:5 *v*/*v*) was added to 50 mg of freeze-dried leaves. Then, samples were mixed thoroughly with 0.2 mL of ferric reagent (2% ferric ammonium sulfate, FeNH_4_(SO_4_)_2_, in 2 M HCl) and incubated in a water bath at 95 °C for 40 min. Absorbance was read at 550 nm. The CTC was calculated using cyanidin chloride as standard and expressed as milligram cyanidin chloride equivalent (CCE) per 100 g DM.

### 2.5. Ultra-High-Performance Liquid Chromatography-Diode Array Detector-Tandem-Mass-Spectrometry (UHPLC-DAD-MS/MS) Analysis of Phenolic Compounds

Samples were analyzed by UHPLC-DAD (Vanquish, Thermo Fischer Scientific, San José, CA, USA) using an Acquity UPLC HSST3 C18 column (100 mm × 1 mm × 1.7 µm, Waters, Milford, MA, USA; 1.7 µm) at 35 °C. The mobile phase consisted of (A) water/formic acid (99/1, *v*/*v*) and (B) acetonitrile/water/formic acid (79.5/19.5/1, *v*/*v*/*v*). The flow rate was 0.22 mL/min. The elution program was as follows: isocratic for 1.5 min with 2% of B, 2–12% of B (1.5–4.5 min), isocratic with 12% of B (4.5–7 min), 12–24% of B (7–12 min), 24–48% of B (12–15 min), 48–60% of B (15–16 min), 60–100% of B (16–17 min).

The UHPLC system was coupled to a high-resolution mass spectrometer (Orbitrap Exploris 480, Thermo Scientific, USA) equipped with an electrospray ionization probe and operated in negative and positive ion modes. The parameters for the ion source were: sheath = 40 a.u., auxiliary gas flow rate = 10 a.u., sweep gas flow rate = 2 a.u., ion transfer tube = 280 °C, vaporizer temperature = 300 °C, mass range = 100–1800 Th, voltage set = 3.5 kV in positive mode and 2.5 KV in negative mode, resolution = 240,000 and 480,000. Phenolic compounds were quantified using the calibration curve of standards based on the UV peak area. Hydroxycinnamic acids were expressed as caffeic acid equivalent at 320 nm. Flavonols and flavones were expressed as quercetin-3-*O*-glucoside at 360 nm. Tryptophan, vanillin, and epigallocatechin gallate were quantified with their own standard at 280 nm. Unknown compounds were expressed as gallic acid (arbitrary choice) at 280 nm.

### 2.6. High-Performance Liquid Chromatography (HPLC) Analysis of β-Carotene and Lutein in Non-Polar Extracts

Carotenoids were analyzed by HPLC using a Thermo Scientific UltiMate 3000 HPLC system (Dionex, Sunnyvale, CA, USA) and a method adapted from that of Courraud et al. (2013) [43]. After filtration through a 0.2 µm polytetrafluoroethylene filter (Macherey-Nagel, Düren, Germany), 20 µL of polar or acetone non-polar extract dilutions was injected into an YMC carotenoid column (250 mm × 4.6 mm I.D., 5 µm particle size; IMC Co., Ltd., Tokyo, Japan). The mobile phase comprised three mixtures (A: methanol and milli-Q water, 60/40 *v*/*v*; B: methanol, methyl tert-butyl-ether and milli-Q water, 28.5/67.5/4 *v*/*v*/*v*; C: ethyl acetate) at a flow rate of 1 mL min^−1^. A gradient program was used. The initial condition was 55% of A/45% of B (0–1 min), then progressively reached 10% of A/90% of B (1–3 min), and 0% of A/100% of B (3–8 min), before remaining at 100% of B (8–10 min), then progressively reached 0% of B/100% of C (10–15 min), remained at 100% of C (15–23 min), and then progressively went back to 55% of A/45% of B (23–26 min), before 9 min of re-equilibration. Carotenoids were detected at 450 nm using a Thermo Scientific Vanquish photodiode array detector. Chromatographic data and UV–visible spectra were integrated using Chromeleon 7 software. Carotenoids were quantified using calibration curves with β-carotene and lutein.

### 2.7. Evaluation of the Antioxidant Capacity of Polar and Non-Polar Extracts 

#### 2.7.1. DPPH Radical Scavenging Assay 

The free radical scavenging capacity of polar extracts toward DPPH radicals was evaluated as described by Hossain et al. (2017) [44] with modifications from the original Blois method [45]. Appropriately diluted polar extracts (60 µL) were mixed with 180 µL of a methanolic solution containing 300 μM DPPH radicals. The mixture was left in the dark for 30 min, and absorbance was measured at 517 nm. Results were determined using the Trolox standard and the antioxidant capacity was expressed as Trolox equivalent (TE) per 100 g DM.

#### 2.7.2. Ferric Reducing/Antioxidant Power (FRAP) Assay

The ferric reducing power of polar extracts was determined according to the method by Benzie and Strain, (1999) [46]. This method is based on the reduction, at low pH, of a colorless ferric-tripyridyltriazine complex (Fe^3+^-TPTZ) to the blue-colored ferrous cation tripyridyltriazine ([Fe (II) (TPTZ) 2]^2+^) by the action of electron-donating antioxidants. The FRAP reagent was prepared with 20 mM ferric chloride (FeCl_3_·6H_2_O) solution, 10 mM 2,4,6-tri(2-pyridyl)-s-triazine (TPTZ) solution in 40 mM HCl and 300 mM acetate buffer at a ratio of 1:1:10 (*v*/*v*/*v*). Then, 30 μL of appropriately diluted samples were added to 90 μL of milliQ water followed by 900 μL of the fresh FRAP reagent in a 96-well plate. After 30 min of incubation at 37 °C, the absorbance of the mixture was read at 593 nm. Results were determined using the Trolox standard and the antioxidant capacity was expressed as TE per 100 g DM.

#### 2.7.3. ABTS Assay

The ABTS assays (polar and non-polar extracts) was performed according to the method by Re et al. (1999) [47] with the slight modifications described by Oboh et al. (2009) [24]. ABTS was dissolved in milliQ water to a 7 mM concentration. ABTS+ radicals were produced by adding 2.45 mM potassium persulfate to the ABTS stock solution and allowing the mixture to stand in the dark at room temperature for 16 h before use. The ABTS+ solution was diluted with ethanol until absorbance of 0.70 (±0.02) at 734 nm. Then, 20 µL of appropriately diluted polar and non-polar extracts were added to 200 µL ABTS+ solution and absorbance was measured at 734 nm after 15 min in dark. Results were determined using the Trolox standard (a water-soluble vitamin E analog) and the antioxidant capacity was expressed as TE per 100 g of DM.

#### 2.7.4. Oxygen Radical Absorbance Capacity (ORAC) Assay

The ORAC assay for polar and non-polar extracts was performed according to the method described by Huang et al. (2002) [21] and Prior et al. (2003) [32] with slight modifications. AAPH was used as a peroxyl radical generator, Trolox as standard, and fluorescein as fluorescent probe. In the ORAC assay, the antioxidant reacts with the peroxyl radical, in competition with fluorescein. The loss of fluorescence is slower, with extracts displaying high antioxidant capacity. All reagents were prepared with 75 mM phosphate buffer (pH 7.4) and the assay was performed in 96-well plates using a fluorescence microplate reader Spark (Tecan Group Ltd., Grödig, Austria. A 25 µL quantity of appropriately diluted polar and non-polar extracts, 150 µL of fluorescein solution (78.75 nM), and 25 µL AAPH (380 mM) were mixed and the fluorescence loss was measured at 37 °C with excitation and emission wavelengths of 485 and 520 nm, respectively, every minute for 1 h. The final ORAC values were calculated using a regression equation between Trolox concentration and the net area under the fluorescein decay curve. The antioxidant capacity was expressed as TE per 100 g DM.

The area under the curve (AUC) was calculated as follows:

AUC = 1 + RFU_1_/RFU_0_ + RFU_2_/RFU_0_ + RFU_3_/RFU_0_ +…+ RFUn/RFU_0_ (1)

where RFU_0_ is the relative fluorescence (in units) at time zero, and RFUn is the relative fluorescence (in units) at the n time point. The net AUC was obtained by subtracting the AUC of the blank from that of the sample.

#### 2.7.5. NO Scavenging Assay

The NO radical scavenging ability of polar and non-polar extracts was evaluated as described by Boora et al. (2014) [48] with modifications from the original method developed in 1864 (Griess, 1965) [49]. Sodium nitroprusside in an aqueous solution and in the light releases NO that interacts with oxygen to produce nitrite ions. Antioxidant molecules compete with oxygen, resulting in a decreased NO production. Briefly, 5 mM of sodium nitroprusside in phosphate buffered saline (PBS) was mixed with each extract (50 µg/mL) and incubated at room temperature for 2 h. The same reaction mixture without the extract but an equivalent amount of PBS served as a positive control. After the incubation period, 100 µL of each sample was mixed with 100 µL of Griess reagent, freshly prepared by mixing equal amounts of sulfanilamide (10 mg/mL) in 5% phosphoric acid and n-(1-naphthyl) ethylenediamine dihydrochloride (1 mg/mL) in milliQ water. After 10 min of incubation in the dark at room temperature, absorbance was measured at 540 nm. Results were determined using the sodium nitrite standard and the extract percentage of nitrite radical inhibition activity was calculated using the following formula:

Nitrite radical inhibition activity (%) = $\frac{A0\;-\;At}{A0}$ × 100 where *A*0 is the absorbance of the positive control and *At* is the absorbance of the sample or standards.

### 2.8. Statistical Analysis

All experiments were performed in triplicate using three different extracts. All data were expressed as the mean ± standard deviation (SD). Data were analyzed with Statgraphics Centurion 19.0 using one-way ANOVA followed by Fisher’s least significant difference (LSD) test. A *p*-value < 0.05 was considered significant. When homogeneity of variance was not respected (Levene’s test), a logarithmic nonparametric test (Games–Howell test) was used. Pearson’s correlation coefficients were calculated to determine the strength of the linear relationships between antioxidant capacity and bioactive compound content.

## 3. Results and Discussion

### 3.1. Polyphenol Content of GLV Samples

- *Total polyphenol, flavonoid and condensed tannin contents*

**Figure 1:**
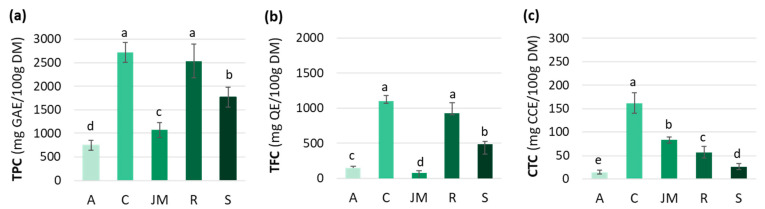
(**a**) Total phenolic content (TPC), (**b**) total flavonoid content (TFC), and (**c**) condensed tannin content (CTC) of amaranth (A), cassava (C), jute mallow (JM), roselle (R) and spinach (S) leaves. GAE: gallic acid equivalent; QE: quercetin equivalent; CCE: cyanidin chloride equivalent; DM: dry matter. Data are expressed as the mean ± SD (TPC (*n* = 33); TFC/CTC (*n* = 9)). Error bars indicate the SD. Different letters above the bars indicate significant differences (*p* < 0.05; LSD or Games–Howell test).

Polyphenols are a diverse and predominant group of secondary metabolites in the plant kingdom. Their distribution and concentration vary widely across and within species, and are influenced by the harvest maturity, season, soil, water stress, and light conditions [50,51]. The TPC, TFC, and CTC varied among the studied African GLV samples (Figure 1). TPC was significantly higher in cassava and roselle leaves (2718 ± 209 and 2534 ± 362 mg GAE/100 g DM, respectively) than in jute mallow (1070 ± 160 mg GAE/100 g DM) and amaranth (745 ± 105 mg GAE/100 g DM) leaves (Figure 1a).

**Figure 2:**
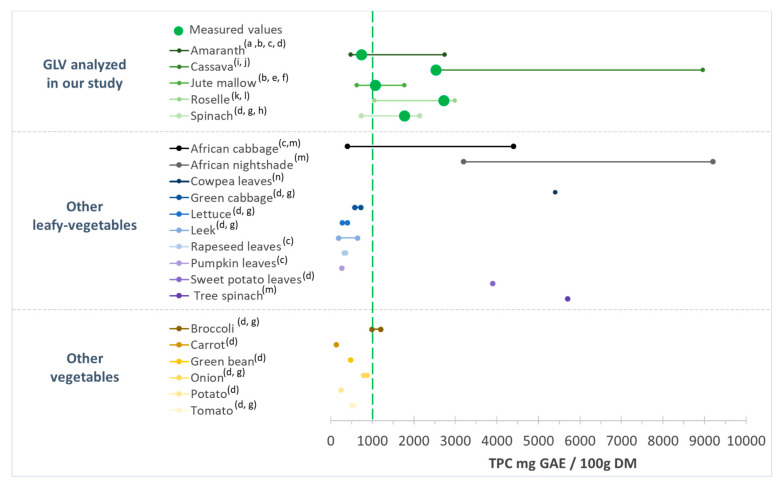
Total phenolic content (TPC) of the green leafy vegetables analyzed in our study in comparison with data from the literature. GAE: gallic acid equivalent; DM: dry matter. (a) [58]; (b) [57]; (c) [14]; (d) [54]; (e) [24]; (f) [59]; (g) [52]; (h) [53]; (i) [50]; (j) [60]; (k) [55]; (l) [51]; (m) [61]; (n) [62].

Spinach TPC content was 1770 ± 213 mg GAE/100 g DM, in agreement with other studies (range: 730 to 2100 mg GAE/100 g DM) (Figure 2) [52,53,54]. According to our results, roselle and cassava leaves had higher TPC than spinach, which gives them an interesting potential. The TPC for roselle leaves was comparable to the values from different countries (e.g., South Africa, Zambia, Nigeria, and Thailand) (1050–2990 mg GAE/100 g DM) [51,55], whereas higher values have been reported for cassava leaves (2500–8960 mg GAE/100 g DM) [50,56]. The TPC values in jute mallow and amaranth leaves were lower than in spinach. Nevertheless, our results remained consistent with data from the literature: 630 to 1770 mg GAE/100 g DM for jute mallow [24,57], and 480 to 2750 mg GAE/100 g DM for amaranth leaves [57,58] (Figure 2).

Differences in TFC among the five GLVs followed the same pattern as for the TPC. The highest TFC-to-TPC ratio was found in cassava leaves (40%), followed by roselle, spinach, and amaranth leaves (37, 28 and 20%), and then jute mallow (7%) (Figure 1b). Overall, the TFC values in our study were lower than those reported by other authors. Jiménez-Aguilar et al. (2017) [58] found TFC values from 490 to 2050 mg QE/100 g for different Amaranthus species, and Ben Yakoub et al. (2018) [16] found from 200 to 400 mg QE/100 g DM for jute mallow leaves, depending on the solvent used during extraction. This great variability is also explained by differences in crop maturity, season, soil, hydric stress, and light. Moreover, the comparison with data from the literature was difficult because the compounds used as standards differed among studies (e.g., quercetin, catechin, rutin).

Comparison with TPC data for different food categories (Figure 2) highlighted that the TPC values of the studied GLV samples were higher than those of other African GLVs (amaranth, African cabbage, squash leaves, and rapeseed leaves) [14] and lower than those of tree spinach, African cabbage, and African nightshade [61,62], depending on the study. Our TPC values were higher than those of other leafy vegetables (green cabbage, lettuce or leek). Only sweet potato leaves had higher TPC values (3900 mg GAE/100 g DM) than our cassava and roselle leaf samples. Similar, our African GLV samples were richer in TPC than other vegetables, such as broccoli, onion, tomato, potato, and carrot [52,54]. Except for amaranth, the studied GLVs had TPC levels > 1000 mg GAE/100 g DM, while most other leafy vegetables and other vegetables used for comparison had TPC levels <1000 mg GAE/100 g DM (Figure 2). Therefore, due to their high TPC, compared with other vegetables, African GLVs could present a special interest for health. 

Condensed tannins (or proanthocyanidins) are catechin oligomers and polymers belonging to the flavanol group. These compounds can form insoluble complexes with carbohydrates and proteins [63]. The CTC highly varied among the studied GLV samples: 162 ± 21 mg CCE/100 g DM in cassava leaves, 83 ± 7 mg CCE/100 g DM in jute mallow leaves, and 57 ± 12 mg CCE/100 g DM in roselle leaves. Spinach and amaranth leaves had much lower CTC (Figure 1c). These results are consistent with data from the literature: 20 to 240 mg/100 g DM for cassava and 4 to 340 mg/100 g DM for jute mallow leaves. However, much higher values were reported for amaranth leaves (11–600 mg/100 g DM). Data for roselle leaves are scarce because many authors have studied the bioactive compounds present in the flowers rather than in the leaves [64]. The CTC values of the four GLVs were lower than those of other leafy vegetables, such as rapeseed leaves, African cabbage, and pumpkin leaves (300, 400 and 1050 mg/100 g DM, respectively) [65,66], but higher than those of vegetables from Mauritania. Indeed, lower CTC values were reported for onion, broccoli, cauliflower and carrot, and CTC was undetectable in all the other vegetables (tomato, lettuce, white cabbage, Chinese cabbage) [67]. Hellström et al. (2009) [68] also did not detect condensed tannins in spinach, carrots, and tomatoes, but found higher values in some fruits, such as blueberries (2160 mg/100 g DM) and cocoa powder (1546 mg/100 g DM). Globally, not many data are available on CTC in GLVs, compared with TPC and TFC. Our analysis showed that cassava leaves are the best source of condensed tannins. Data from the literature on the content and composition of dietary tannins are incomplete and insufficient to determine the daily dietary intakes. Two studies estimated the daily intake for the general population in the United States and in Spain (50–500 mg/day/person) [63,69]. In recent years, particular attention has been paid to condensed tannins due to their immunomodulatory, anti-inflammatory, anticancer, antioxidant, cardio-protective, and antithrombotic properties. Therefore, the consumption of cassava leaves might significantly contribute to the tannin intake of people in sub-Saharan Africa.

- *Identification of phenolic compounds*

**Figure 3:**
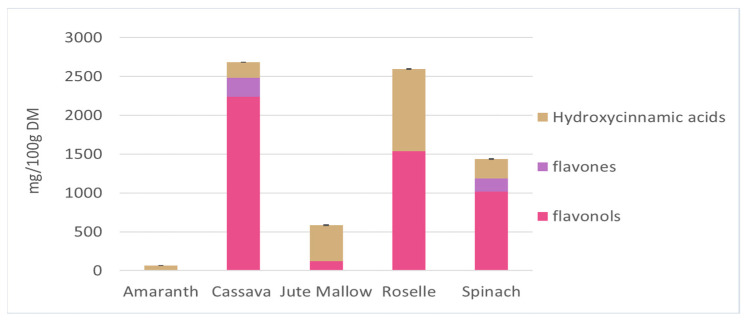
Different phenolic compounds from the three main classes of hydroxycinnamic acids, flavonols, and flavones identified in polar extracts of amaranth, cassava, jute mallow, roselle, and spinach leaves. Hydroxycinnamic acids are expressed as caffeic acid equivalent and flavonols/flavones as quercetin-3-*O*-glucoside equivalent. Data are the mean ± SD of three replicates. Error bars indicate the SD of the sum of the phenolic compounds classes.

The main soluble phenolic compounds in amaranth, cassava, jute mallow, roselle, and spinach leaves were characterized by UHPLC-DAD-MS/MS based on the following features: mass spectra, accurate mass, characteristic fragmentation, UV spectrum, and characteristic retention time. This analysis led to the tentative identification of 67 compounds belonging to several chemical classes: hydroxycinnamic acids, flavonols (glycosides, cinnamoyl glycosides, and aglycones) and flavones based on their UV–visible spectra (Table S1). The chromatograms of these five GLV polar extracts are shown in Figure S1 and the identity of the peaks are listed in Table S1. Most of the identified hydroxycinnamic acids have been previously described. Clarifications were added about isomers (position of substituents, or cis vs. trans isomers) based on fragmentation spectra [70,71,72]. Only one hydroxycinnamic acid has not described previously: in spinach leaves, the peak at 5.92 min could be a coumaric acid derivative, probably a hexoside (C_6_H_10_O_5_), according to the HRMS data. Some flavonols and flavones were tentatively identified for the first time: quercetin-*O*-hexose-dideoxyhexose, quercetin-*O*-hexose-deoxyhexose and kaempferol-*O*-hexose-deoxyhexose in roselle leaves, and isorhamnetin-*O*-hexoside in cassava leaves. In roselle leaves, the compounds at 13.85 and 16.69 min had molecular ions at *m/z* 609.125 and that they yielded fragments at *m/z* 300.027 and 463.087 might indicate the presence of a quercetin derivative with hexoside moieties. The identification of kaempferol 3-*O*-coumaroylglucoside in a previous work [73] suggests that these compounds could be two quercetin 3-*O*-p-coumaroylglucoside isomers. Figure 3 presents the concentration of the three main classes of phenolic compounds (hydroxycinnamic acids, flavonols, and flavones) identified in GLV polar extracts. Cassava and roselle leaves had significantly higher phenolic compound contents than the other GLVs (2683 and 2592 mg/100 g DM, respectively). These results corroborate the previous results on TPC. Flavonols were the main class found in cassava and roselle leaves (90 and 60%, respectively). Roselle leaves contained the greatest diversity of phenolic compounds (*n* = 26 molecules identified). The three main compounds were quercetin 3-*O*-rutinoside, kaempferol 3-*O*-rutinoside (flavonols), and 3-caffeoylquinic acid (hydroxycinnamic acid). These compounds represented 75% of the total phenolic compounds in roselle leaves. Quercetin 3-*O*-rutinoside (1527.5 mg/100 g DM) and kaempferol 3-*O*-rutinoside (660 mg/100 g DM) were also found in cassava leaves (80% of the total phenolic compounds). Spinach leaves contained mainly flavonols (70%), mostly compounds derived from patuletin and spinacetin. Hydroxycinnamic acids were the main polyphenol class in jute mallow leaves (80%), and the main compound was 3,5-dicaffeoylquinic acid (56% of the TPC). However, this compound was not found in any other GLVs. Amaranth leaves had a very low content in phenolic compounds, only from the hydroxycinnamic acid family (coumaric, ferulic and feruloyl glucaric acid).

All these data highlight the wide quantitative and qualitative variability in phenolic compounds present in the African GLVs tested, with a particularly high TPC and flavonoid content in cassava and roselle leaves. The analysis of the phenolic compound profile allowed us to identify several compounds, such as rutin, quercetin, and kaempferol, for which various biological effects have been reported, particularly antioxidant capacity [55,74]. When consumed regularly, these GLVs, especially cassava and roselle leaves, could significantly contribute to the polyphenol and flavonoid intake. However, no recommendation currently exists on the daily intake levels of polyphenols due to the lack of sufficient data [75]. A systematic review of data from the literature of the last 10 years on polyphenol intake and its association with specific disease markers [76] was made and an approximate mean intake of ~900 mg/day was proposed despite the large data heterogeneity. Most studies were performed in Europe, North America, and Asia in adult populations and the main scientific databases used for the estimation of polyphenol intake were the United States Department of Agriculture (USDA) and Phenol-Explorer databases [77]. However, more food databases should be developed to consolidate information on different polyphenol subcategories [76].

### 3.2. Carotenoid Content of GLVs

**Figure 4:**
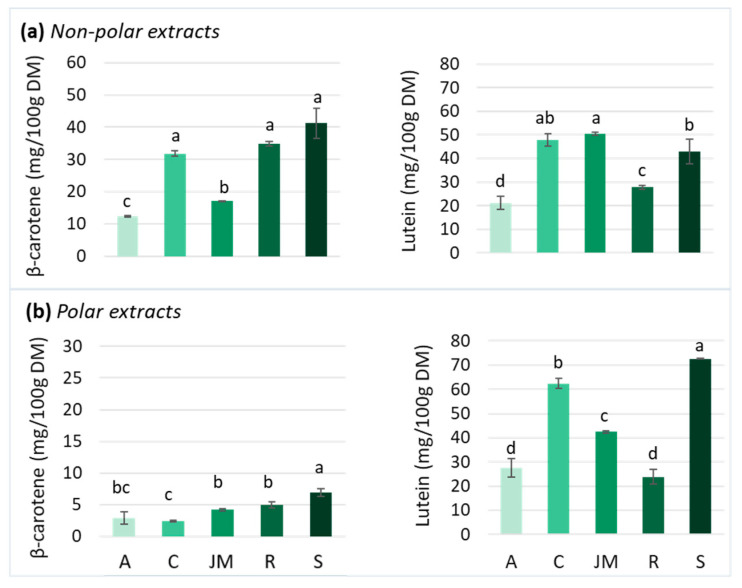
β-carotene and lutein contents in (**a**) non-polar and (**b**) polar extracts of amaranth (A), cassava (C), jute mallow (JM), roselle (R), and spinach (S) leaves. DM: dry matter. Data are the mean ± SD (n = 3). Error bars indicate the SD. Different letters above the bars indicate significant differences (*p* < 0.05; LSD or Games–Howell test).

Carotenoids are particularly abundant in most yellow-orange fruits and vegetables and also in GLVs, although they are masked by chlorophylls [78]. Carotenoids are usually divided into two groups: oxygen-devoid carotenes and oxygen-containing xanthophylls, such as lutein. Several carotenoids are vitamin A precursors: β-carotene, α-carotene, and β-cryptoxanthin. Due to conjugated double bonds, carotenoids are radical scavengers and singlet oxygen quenchers, thereby reducing oxidative stress and chronic inflammation [30]. Carotenoids are the most abundant lipid-soluble phytochemicals in fruits and vegetables and they can act on intracellular signaling cascades, influencing cytokine gene expression and protein translation [30]. Lutein (nearly 45%) and β-carotene (25–30%) are the predominant carotenoids found in GLVs, such as spinach, kale, amaranth, cassava, and moringa leaves [79,80] although their concentration varies considerably among vegetables. Therefore, this study focused on β-carotene and lutein content in non-polar and polar extracts. The chromatograms of the carotenoids of non-polar and polar extracts of each GLVs are presented in Figure S2 and Figure S3, respectively. Among the studied GLV samples, spinach, cassava, and roselle leaves presented β-carotene contents of ~40 mg/100 g DM, i.e., more than twice as high as those found in jute mallow and amaranth leaves (around 15 mg/100 g DM) (Figure 4a). Due to its hydrophobic nature, β-carotene is present in only low amounts in polar extracts (Figure 4b).

**Figure 5:**
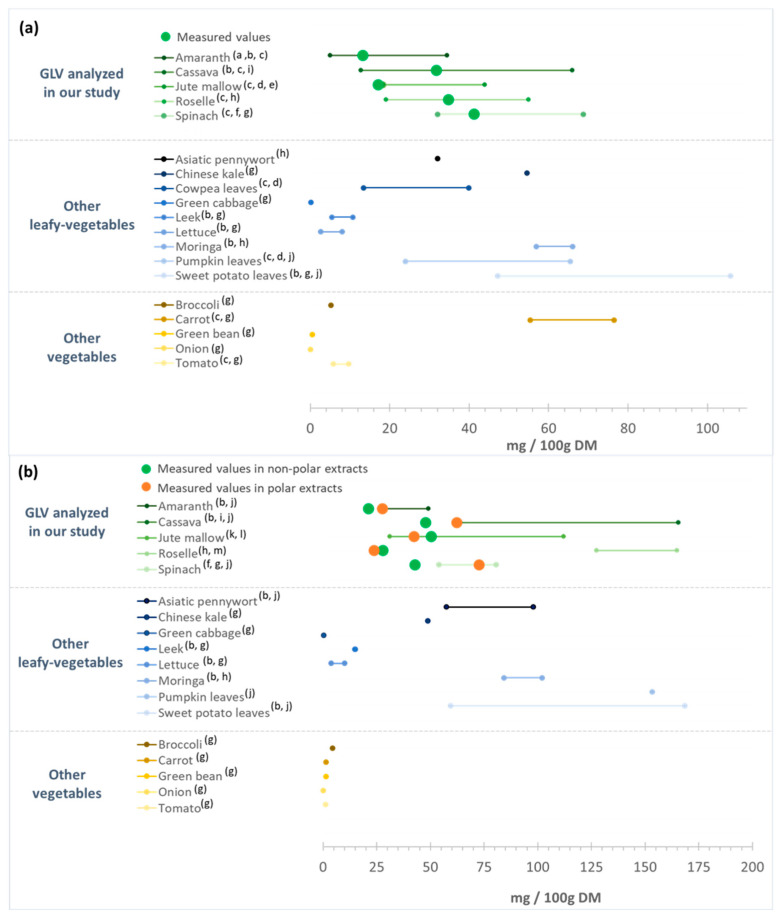
(**a**) β-carotene and (**b**) lutein content of the green leafy vegetables analyzed in our study in comparison with data from the literature. DM: dry matter. (a) [84]; (b) [80]; (c) [81]; (d) [83]; (e) [15]; (f) [53]; (g) [54]; (h) [82]; (i) [60]; (j) [19]; (k) [86]; (l) [85]; (m) [87].

Previous studies reported similar β-carotene contents in spinach [53,54], roselle [81,82] and cassava leaves [60,81], and also in jute mallow [15,83] and amaranth [80,84], although the present values for these last two were in the lower range (Figure 5a). Like for polyphenols, the carotenoid content in GLVs may vary according to the species, cultivation areas, use of fertilizers, and storage conditions. Several GLVs, such as cowpea and pumpkin leaves, exhibit β-carotene content in the same range as those found in the five GLVs tested, whereas sweet potato leaves and moringa leaves seem to have higher β-carotene contents [19,54,80,81,83]. Some leafy vegetables (green cabbage, leek, and lettuce) have lower β-carotene content than the GLVs tested, while non-leafy vegetables (broccoli, green bean, onion, and tomato) had a much lower β-carotene content [54,81]. Only carrots have a higher β-carotene content (55–76 mg/100 g DM) than those found in most GLVs, except pumpkin leaves (Figure 5a). 

Lutein content was higher in non-polar extracts of jute mallow, cassava, and spinach leaves than of roselle and amaranth leaves (Figure 4a). Due to the presence of two hydroxyl groups, lutein exhibits a hydrophilic nature. Consequently, lutein contents were also measured in the polar extracts. Amaranth, cassava, and spinach leaves presented higher content in polar than in non-polar extracts, unlike roselle and jute mallow leaves (Figure 4b). These results were consistent with other studies that reported lutein levels ranging from 31 to 112 mg/100 g DM in jute mallow leaves [85,86] and from 54 to 81 mg/100 g DM in spinach [53,54] (Figure 5b). However, the values for amaranth and cassava leaves were in the lower range [60,80]. Moreover, lutein concentration in roselle leaves was six times higher than what described previously [82,87].

Lutein content in the studied African GLVs was relatively comparable with that of other GLVs (Figure 5b). Some leafy vegetables have higher lutein content, such as sweet potato leaves and pumpkin leaves (60 to 170 mg/100 g DM [54,80]. Conversely, green cabbage, leek, and lettuce are poor in lutein (0.3–15 mg/100 g DM). Our four GLVs displayed much higher lutein content than other vegetables, such as broccoli, carrot, green bean, onion, and tomato (0.1 to 4.5 mg/100 g DM) [54]. This confirmed the rather high lutein content of cassava and jute mallow leaves, comparable to that of spinach and other African leafy vegetables. Lutein is essential for the antioxidant capacity of these vegetable, acting to quench free reactive radicals to prevent chronic inflammation and chronic diseases [88]. As observed for polyphenols, carotenoid content hugely varied according to the species. Overall, spinach showed the highest carotenoid content (sum of β-carotene and lutein), in agreement with Isabelle et al. (2010) [54]. Cassava, roselle, and jute mallow leaves had carotenoid contents similar to spinach and thus are a good source of provitamin A (β-carotene). High uptake of provitamin A could prevent vitamin A deficiency and protect against oxidative damage. It is important to note that data on the carotenoid content of roselle leaves are particularly scarce and should not be confused with those obtained on calyces (or flowers). Indeed, roselle calyces are widely used to prepare a decoction (e.g., bissap) and are rich in bioactive compounds (anthocyanin, β-carotene, lycopene, and lutein).

### 3.3. Antioxidant Capacity of Polar and Non-Polar GLV Extracts

Antioxidants are molecules that can neutralize free radicals by accepting or donating electron(s), thus reducing their capacity to cause cellular damage. A single antioxidant can exhibit different mechanisms of action, such as ROS/RNS scavenging to stop radical chain reactions, transition metal chelation, oxidative enzyme inhibition, or activation of antioxidative enzyme cofactors [21]. As different antioxidant compounds may act in vivo through different mechanisms, no single method can fully evaluate the total antioxidant capacity of foods. Therefore, five assays, based on different mechanisms (see Introduction), were used to obtain robust data on the antioxidant capacity of the selected African GLVs. Table 1 shows the antioxidant capacity values of the polar and non-polar extracts in terms of the method used, their ranking order for each assay, and the mean rank. In polar extracts, roselle and cassava leaves presented significantly higher antioxidant capacity, assessed with the FRAP and DPPH assays, than the other leaves. Amaranth leaves exhibited the lowest activity (5 to 10 times lower, respectively, than roselle and cassava leaves). Cassava leaves also presented the highest antioxidant capacity with the ORAC and NO scavenging methods, followed by roselle leaves. The amaranth value remained the lowest. Conversely, with the ABTS assay, the greatest value was obtained for amaranth and cassava (around 8.5 mmol TE/100 g DM), followed by spinach (6.4 ± 0.3 mmol TE/100 g DM), roselle (5.8 ± 0.5 mmol TE/100 g DM), and then jute mallow leaves (3.7 ± 0.6 mmol TE/100 g DM). This discrepancy concerning amaranth leaves could be due to the presence of a compound that interferes with this assay rather than to the presence of a compound giving this leaf a higher ABTS value. Indeed, the presence of feruloyl glucaric acid (the main compound in our polar extract of amaranth leaves) is not necessarily correlated with a high antioxidant capacity value [89]. In summary, the polar extract from cassava leaves exhibited the highest antioxidant activity, followed by roselle leaves (Table 1). Therefore, these two GLVs are particularly interesting in preventing oxidative stress. The amaranth leaf polar extract was ranked last.

The antioxidant capacity of non-polar extracts was assessed only with the ABTS, ORAC, and NO scavenging methods, which are applicable to lipophilic extracts (Table 1). The FRAP and DPPH assays, which assess the antioxidant capacity of hydrophilic samples, could not be used because (i) the FRAP assay requires an aqueous medium and (ii) the spectrophotometric measurements for DPPH can be affected by carotenoids (Prior et al., 2005). With the ABTS assay, antioxidant capacity values ranged from 2.3 to 0.5 mmol TE/100 g DM, and spinach leaves presented the highest values. With the ORAC assay, values varied from 3.7 to 1.1 mmol TE/100 g DM, and roselle and cassava leaves presented the highest values. With the NO scavenging assay, jute mallow and spinach leaves were the most effective (>55% inhibition) (Table 1). Thus, the spinach leaf non-polar extract was the highest ABTS radical scavenger, possibly due to its high β-carotene content. On the other hand, the jute mallow leaf non-polar extract was the highest scavenger of the NO radical, possibly due to its high lutein content. The non-polar roselle leaf extract was the most effective at scavenging peroxyl radicals. When looking at the mean rank, the non-polar extract of amaranth leaves remained in the last position, whereas the spinach, jute mallow, and cassava leaf non-polar extracts were the most effective.

The antioxidant capacity of vegetables is usually measured from polar extracts. Compared with other vegetables, spinach polar extracts (i.e., the reference) exhibited the highest antioxidant capacity according to Proteggente et al. (2002) and Isabelle et al. (2010) [52,54], with values similar to those obtained in the present study using the ABTS, ORAC, and FRAP assays. 

Our results show that roselle and cassava leaf polar extracts have higher antioxidant capacity than spinach. Similar values were previously reported for roselle leaves with the ABTS assay [55] and the FRAP and DPPH assays [90]. Conversely, amaranth leaf polar extracts had lower antioxidant capacity than spinach, but our results are not consistent with the literature. For example, Jimenez-Aguilar et al. (2017) [58] found values two to five times higher than our results with the ORAC method, and Catarino et al. (2019) [90] found values 6 to 20 times higher with the FRAP and DPPH assays. The cultivar, harvest maturity, growing conditions (location, soil, climate, agriculture practices) and storage conditions could explain these major differences within the same species. For example, when stored at 4 and 20 °C, the antioxidant capacity of spinach decreased compared to the initial values, while levels remained constant when continually stored at −20 °C [91].

Comparison with the antioxidant capacity (ORAC assay) of the 66 vegetables analyzed by Isabelle et al. (2010) [54] indicated that the values of our amaranth, jute mallow, and spinach leaves were similar to those of most leafy vegetables, while cassava and roselle leaves had higher (1.5 to 8 times) values, except relative to sweet potato and wolfberry leaves. Moreover, their antioxidant capacity values were much higher compared with non-leafy vegetables, such as broccoli, carrot, green bean, onion, and tomato: up to 20 times higher for cassava and roselle leaves [54]. Our study confirmed that cassava and roselle leaves have rather interesting antioxidant capacities, higher than spinach, which is known to have a great antioxidant capacity. The methods used in this work indicated that they contain compounds that can scavenge free cation radicals (ABTS^•+^) and peroxyl radicals, thereby inhibiting lipid oxidation via a chain-breaking reaction and also reducing oxidants (ferric ions). For all GLVs, the antioxidant capacity of polar extracts, which contain mostly polyphenols and flavonoids, was significantly higher than that of non-polar extracts, which contain mainly carotenoids, in agreement with other studies [23,24]. The high antioxidant capacity of these batches of African GLVs suggests that they could play a role in preventing oxidative stress and chronic inflammation.

### 3.4. Correlation between Antioxidant Capacity and Bioactive Compounds

**Figure 6:**
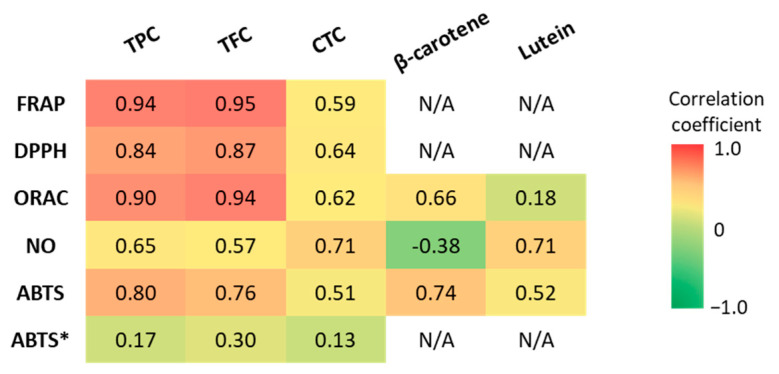
Pearson’s correlation coefficients (*r*) between antioxidant capacity (FRAP, DPPH, ORAC, ABTS, NO scavenging assays) and TPC, TFC, CTC, β-carotene, and lutein content. TPC: total polyphenol content, TFC: total flavonoid content, CTC: condensed tannin content. ABTS*: including the values for amaranth leaf extracts.

Pearson’s correlation coefficients between the antioxidant capacity values and bioactive compound content were determined to identify the main GLV compounds that contributed to the observed antioxidant capacity. The correlation strengths were classified as “very weak” (r = 0.00–0.19), “weak” (r = 0.20–0.39), “moderate” (r = 0.40–0.59), “strong” (r = 0.60–0.79), and “very strong” (r = 0.80–1.0) [92] (Figure 6). The TPC and TFC displayed strong to very strong correlations (*p* < 0.05) with the antioxidant capacity evaluated with the FRAP, ORAC, DPPH, and ABTS methods. However, when the data for amaranth leaf extracts obtained with the ABTS method were taken into account, the correlation with TPC and TFC disappeared. Indeed, the TPC was low in amaranth leaves, whereas the antioxidant capacity measured with the ABTS method was high. When evaluated with the NO scavenging method, antioxidant capacity was strongly correlated with the TPC (r = 0.65), but only moderately with the TFC (r = 0.57). Generally, the antioxidant capacity was moderately correlated with the CTC (r = 0.51 to 0.71 in function of the method). This could be explained by the low CTC in our GLV polar extracts.

These findings highlight the contribution of polyphenols and flavonoids to the in vitro antioxidant capacity of GLVs by scavenging free radicals (DPPH, ABTS, ROO^•^) as well as reducing oxidants (ferric ions) and the need to use different antioxidant assays. Indeed, recent studies have shown that flavonoids and polyphenols contribute significantly to the total antioxidant capacity of vegetables [92]. Previous studies reported very good correlations between GLV antioxidant capacity and TPC [57,78,93] and TFC [94]. Crude extracts of polyphenols from jute mallow leaves containing mainly chlorogenic acid (5-caffeoylquinic acid) and rutin (quercetin-3-*O*-rutinoside) have antioxidant capacity due to their ability to scavenge the DPPH radical [74]. These two molecules were also identified in our cassava and roselle leaf polar extracts. Moreover, chlorogenic acid, quercetin and kaempferol scavenge ABTS radical cations. They were previously found in methanolic extracts of roselle leaves and also in cassava and roselle leaf extracts [55]. The high antioxidant capacity of cassava and roselle leaf polar extracts may be due to their high polyphenol content. Indeed, they are the best scavengers of DPPH, NO, and peroxyl radicals and the GLVs with the highest ferric-ion-reducing ability.

The antioxidant capacity of non-polar extracts (ABTS and ORAC methods) was strongly correlated (*p* < 0.05) with β-carotene content (r = 0.74 and r = 0.66, respectively), and more weakly with lutein content (r = 0.52 and 0.18, respectively). However, the ability to scavenge NO^•^ was strongly correlated only with lutein content (r = 0.71), because the correlation with β-carotene was negative (r = −0.38). These results show that β-carotene is most likely involved in the scavenging of free radicals (ABTS assay) and peroxyl radicals (ROO^•^) (ORAC assay). Peroxyl radicals can be generated during lipid peroxidation and then propagate peroxidation, which is initiated by the abstraction of an H atom from polyunsaturated fatty acids. On the other hand, lutein can scavenge NO^•^. An excess of NO^•^ is cytotoxic. It might combine with O2^•−^ to form peroxynitrite (ONOO^−^). Carotenoids have a common structure (basic structure of at least 40 carbons with a conjugated double bond system) that gives them the ability to scavenge free radicals. However, due to their differences (e.g., presence or absence of oxygen), they do not act in the same way and can scavenge different free radicals [95]. Some studies analyzed the correlation between antioxidant capacity and carotenoid content in fruits and vegetables. Sarker et al. (2018) [96] found a strong correlation of total carotenoid and β-carotene content with the antioxidant capacity (ABTS method) of amaranth leaves (r = 0.96 and r = 0.92, respectively; slightly higher but consistent with our results). The same authors analyzed 12 amaranth genotypes and found that total carotenoids were strongly correlated with the ABTS assay results [18]. These findings indicate that an increase in carotenoid content was directly related to the antioxidant capacity increment (ABTS method). Compared with our result, better correlations were observed between antioxidant capacity (ORAC assay) and lutein content (r = 0.57) in lipophilic extracts of broccoli [97]. Conversely, the ORAC assay results were weakly correlated with the β-carotene content (R^2^ = 0.48) of sweet potato lipophilic extracts [98]. Other carotenoids, such as violaxanthin and neoxanthin, were not measured in our study, but they also may contribute to the antioxidant capacity of lipophilic components. This could explain the lower correlations of antioxidant capacity with carotenoid content (β-carotene plus lutein) in non-polar GLV extracts than with TPC and TFC in polar extracts [79,80]. In addition, as the main antioxidant effect of carotenoids is due to the neutralization of singlet oxygen, other assays to evaluate the antioxidant capacity, such as the singlet oxygen (^1^O_2_) quenching and β-carotene bleaching assays, could be required [95]. Because of their antioxidant properties, a diet rich in carotenoids is associated with a reduced risk of several disorders caused by oxidative stress, such as some cancer types, cardiovascular diseases, and macular degeneration [79]. The high antioxidant capacity of spinach and manioc leaves could be due to their high total carotenoid content. Indeed, several studies have shown that β-carotene is involved in the scavenging of free radicals and peroxyl radicals (ROO^•^) [99]. The present study revealed that the antioxidant capacity of GLVs is more correlated with the TFC and TPC than with β-carotene and lutein contents, which are nonetheless significant. Cassava and roselle leaves emerge as valuable sources of antioxidants.

## 4. Conclusions

The bioactive compound contents and antioxidant capacity of the five GLVs varied widely according to the species, the method used, and the type of extract, but were generally higher than those of other vegetable types. The antioxidant capacity of GLVs was higher in polar than in non-polar extracts, and was strongly positively correlated with TPC and TFC (r > 0.8), suggesting a preponderant role of polar compounds. Nevertheless, GLV non-polar extracts exhibited significant antioxidant capacity, likely due to the presence of carotenoids (β-carotene or lutein). Antioxidant capacity values varied depending on the in vitro assay, suggesting different antioxidant mechanisms. This study highlights the need of using different antioxidant assays to evaluate the antioxidant capacity. Overall, cassava and roselle leaves exhibited a higher antioxidant capacity than spinach, which is known to have an important antioxidant effect. Therefore, in addition to their interesting nutritional value, they may offer additional health benefits due to their high contents of bioactive compounds involved in preventing oxidative stress. More studies are required to assess the effectiveness of the antioxidant and possibly anti-inflammatory effects related to the consumption of leafy vegetables on gut inflammatory status.

## Figures and Tables

**Table 1:** Antioxidant capacity of polar and non-polar extracts.

		DPPH		FRAP		ABTS		ORAC		NO Scavenging	
Green Leafy Vegetables	Mean Rank *	Value	Rank	Value	Rank	Value	Rank	Value	Rank	Value	Rank
		(mmol TE/100 g DM)								(NO inhibition (%)) ^§^	
**Polar extracts**											
**Amaranth**	**5**	0.8 ± 0.1 ^d^	5	4.3 ± 0.4 ^d^	5	8.9 ± 0.2 ^a^	1	9.2 ± 0.9 ^d^	4	41 ± 1.3 ^d^	5
**Cassava**	**1**	8.4 ± 0.4 ^a^	1	20 ± 1.4 ^a^	1	8.3 ± 0.5 ^a^	1	47 ± 3.5 ^a^	1	76 ± 2.0 ^a^	1
**Jute mallow**	**4**	3.5 ± 0.3 ^c^	3	7.2 ± 0.2 ^c^	4	3.7 ± 0.6 ^c^	5	13 ± 1.8 ^d^	4	72 ± 1.1 ^b^	2
**Roselle**	**2**	8.7 ± 0.6 ^a^	1	20 ± 1.3 ^a^	1	5.8 ± 0.5 ^b^	3	34 ± 1.3 ^b^	2	72 ± 0.8 ^b^	2
**Spinach**	**3**	2.5 ± 0.5 ^b^	4	10 ± 0.4 ^b^	3	6.4 ± 0.3 ^b^	3	19 ± 2.6 ^c^	3	59 ± 2.9 ^c^	4
**Non-polar extracts**											
**Amaranth**	**5**	N/A				0.5 ± 0.04 ^c^	5	1.1 ± 0.1 ^d^	5	43 ± 1.4 ^d^	4
**Cassava**	**3**	N/A				1.3 ± 0.1 ^b^	2	3.1 ± 0.3 ^b^	2	47 ± 0.6 ^c^	3
**Jute mallow**	**1**	N/A				1.1 ± 0.1 ^b^	2	2.1 ± 0.2 ^c^	3	58 ± 0.7 ^a^	1
**Roselle**	**3**	N/A				1.1 ± 0.1 ^b^	2	3.7 ± 0.3 ^a^	1	42 ± 0.9 ^d^	4
**Spinach**	**1**	N/A				2.9 ± 0.1 ^a^	1	2.4 ± 0.2 ^c^	3	55 ± 0.2 ^b^	2

Values are expressed as mean ± SD (n = 9). Different letters in the same column indicate significant differences (*p* < 0.05; one-way ANOVA followed by the post hoc Fisher’s LSD test or the Games–Howell test). DM: dry matter; N/A: not applicable; TE: Trolox equivalent. * ranking from the more antioxidant (1, dark green), to the less antioxidant (5, red), mean rank is the average of the ranks obtained with each method for each LV; ^§^ percentage of NO^•^ radical inhibition by 50 µg/mL of dry extracts.

## Data Availability

The data are contained within the article.

## Supplementary Materials

The following supporting information can be downloaded at: https://www.mdpi.com/article/10.3390/antiox12091726/s1; Table S1: Identification of phenolic compounds detected in polar extracts of amaranth, cassava, jute mallow, roselle and spinach leaves. Figure S1: Chromatograms of phenolic compounds in polar extracts of amaranth (A), cassava (B), jute mallow (C), roselle (D) and spinach (E) leaves. The identity of the peaks are listed in Table S1; Figure S2: HPLC chromatograms of carotenoids in non-polar extracts of amaranth (A), cassava (B), jute mallow (C), roselle (D) and spinach (E) leaves, at 450 nm; Figure S3: HPLC chromatograms of carotenoids in polar extracts of amaranth (A), cassava (B), jute mallow (C), roselle (D) and spinach (E) leaves, at 450 nm. References [17,70,71,72,73,84,100,101,102,103,104,105,106,107,108,109,110,111,112,113,114,115] are cited in the supplementary materials (Table S1).
