# Supplementary material for: Antioxidant Capacity of Polar and Non-Polar Extracts of Four African Green Leafy Vegetables and Correlation with Polyphenol and Carotenoid Contents
Source: Antioxidants (Basel). 2023 Sep 6;12(9):1726. doi: 10.3390/antiox12091726 (PMC10525563; doi:10.3390/antiox12091726)
Supplement: Supplementary file 1 [file antioxidants-12-01726-s001.zip › antioxidants-2543162-supplementary/Figure S1_antioxidants-2543162_Chromatograms of phenolic compounds.pdf]

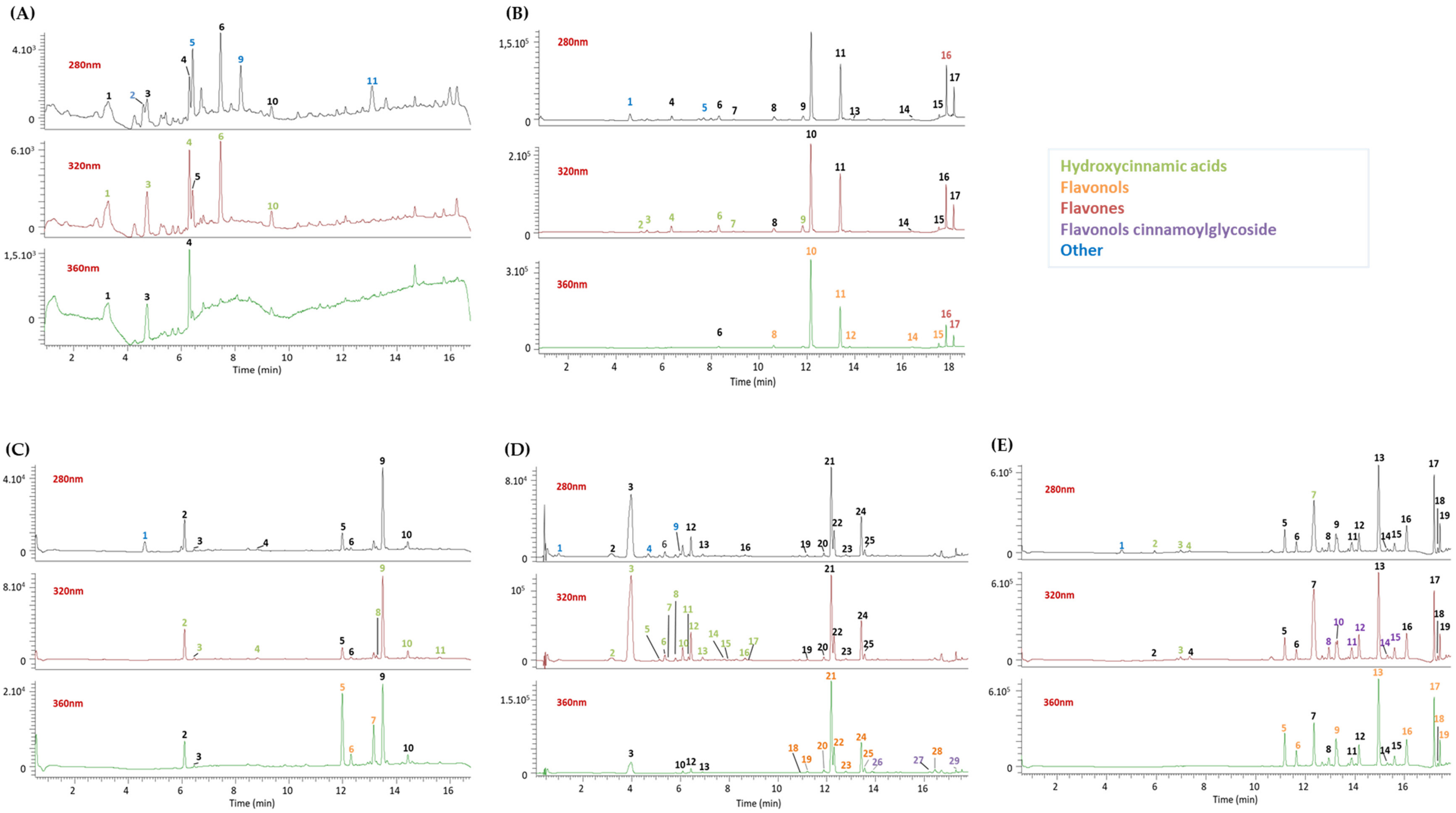

Figure S1. Chromatograms of phenolic compounds in polar extracts of amaranth (A), cassava (B), jute mallow (C), roselle (D) and spinach (E) leaves. The identity of the peaks are listed in Table S1.
