# Supplementary material for: Antioxidant Capacity of Polar and Non-Polar Extracts of Four African Green Leafy Vegetables and Correlation with Polyphenol and Carotenoid Contents
Source: Antioxidants (Basel). 2023 Sep 6;12(9):1726. doi: 10.3390/antiox12091726 (PMC10525563; doi:10.3390/antiox12091726)
Supplement: Supplementary file 1 [file antioxidants-12-01726-s001.zip › antioxidants-2543162-supplementary/Figure S3_antioxidants-2543162_Chromatograms of carotenoids_polar extracts.pdf]

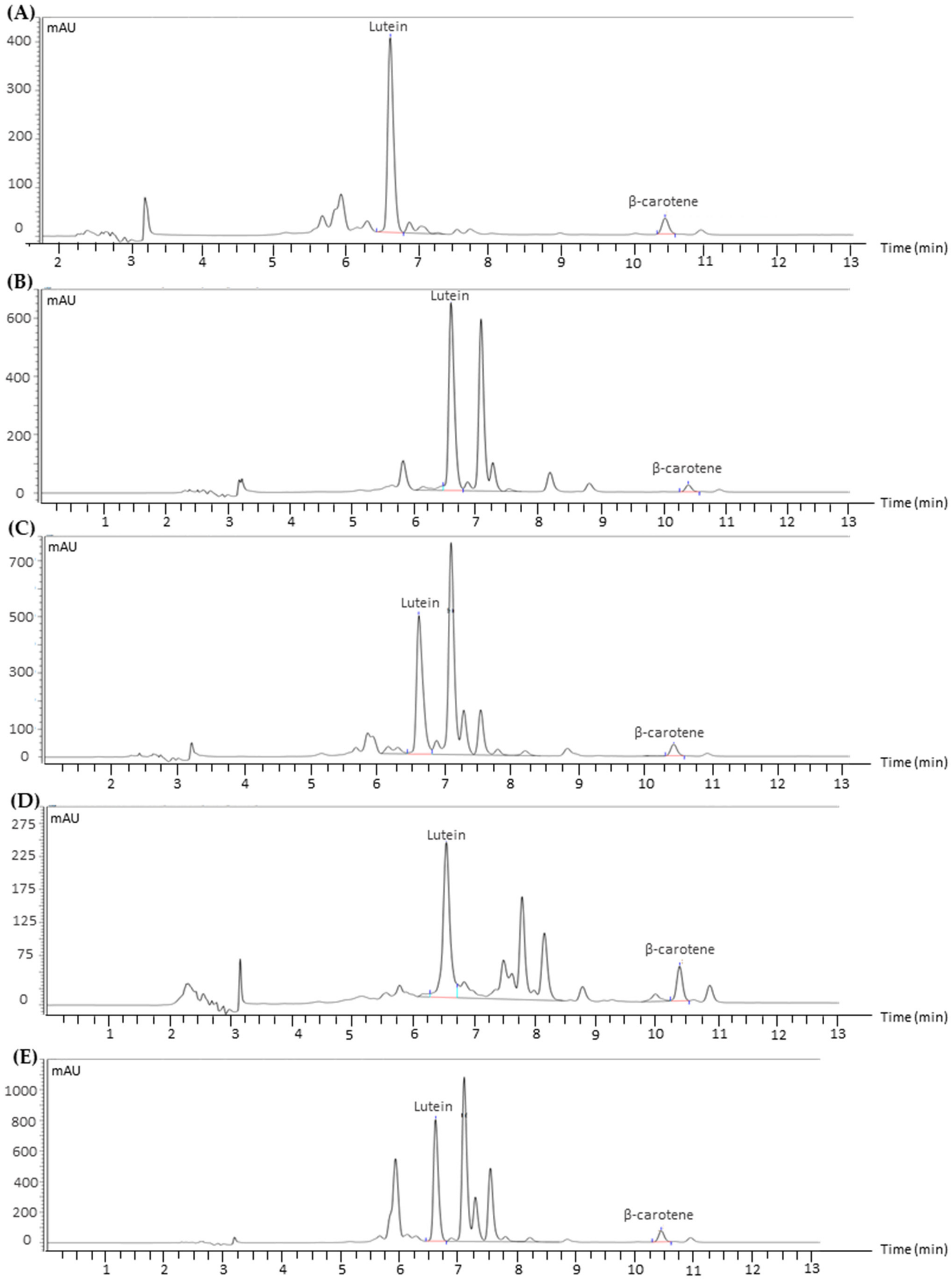

Figure S3. HPLC chromatograms of carotenoids in polar extracts of amaranth (A), cassava (B), jute mallow (C), roselle (D) and spinach (E) leaves, at 450 nm
