# Supplementary material for: Antioxidant Capacity of Polar and Non-Polar Extracts of Four African Green Leafy Vegetables and Correlation with Polyphenol and Carotenoid Contents
Source: Antioxidants (Basel). 2023 Sep 6;12(9):1726. doi: 10.3390/antiox12091726 (PMC10525563; doi:10.3390/antiox12091726)
Supplement: Supplementary file 1 [file antioxidants-12-01726-s001.zip › antioxidants-2543162-supplementary/Table S1_antioxidants-2543162_Identification of phenolic compounds.pdf]

Table S1. Identification of phenolic compounds detected in polar extracts of amaranth, cassava, jute mallow, roselle and spinach leaves

Peacks	Rt (min)	λ max (nm)	Ionization mode	m/z	Main fragments (% intensity)	Molecular formula	Error (ppm)	Tentative identification	References	Conc. (mg/L)	*RSD* (%)	Class	Conc. (mg/L)
**AMARANTH**													
1	3.28	310sh-330	-	385.0764	85.0295(100); 129.0193(20); 191.0197(20); 147.0299(10); 209.0302(10)	C16H17P11	-1.7	Feruloylglucaric ac. I	Neugart 2017[a]	7.09	*0.93*	Hydroxycinnamic acids	22.21
3	4.72	294sh-326	-	385.0764	85.0295(100); 191.0197(20); 209.0302(15); 147.0298(10); 129.0193(10)	C16H17O11		Feruloylglucaric ac. II	Neugart 2017[a]	5.68	*1.23*		
4	6.3	294sh-330	-	385.0764	85.0295(100); 191.0197(25); 209.0303(15); 129.0193(15); 147.0299(15)	C16H17O11	-1.6	Feruloylglucaric ac. III	Neugart 2017[a]	5.51	*2.58*		
6	7.45	310	+	165.0547	147.0441	C9H9O3	0.2	Coumaric ac.	Standard; Klimczak 2002[b]	3.05	*2.45*		
10	9.34	293-322	+	195.0652		C10H11O4	0.1	Ferulic ac.	Standard; Klimczak 2002[b]	0.88	*0.80*		
2	4.59	278	+	205.0971	188.0706(100); 146.0600(20)	C11H13O2N2	-0.1	Tryptophan	standard	3.74	*7.42*	Others	13.24
5	6.41	278-306	+	153.0547	125.0598(100); 111.0441(100); 93.0335(80); 65.0386(50)	C8H9O3	0.8	Vanillin	standard	4.97	*1.16*		
9	8.2	282	/					NI		NQ	/		
11	13.07	270	-	289.111	96.9601	/	/	NI		4.53	*2.61*		
												**TOTAL**	**35.45**
**CASSAVA**													
2	5.07	305	-	337.0929	163.04(100); 191.0560(40); 119.0502(20)	C16H17O8	0.14	*cis*-3-Coumaroylquinic ac.	Perez-Fons 2019[c]; Jaiswal 2011[d]	2.29	*4.87*	Hydroxycinnamic acids	56.23
3	5.3	293sh-310	-	337.093	163.04(100); 119.0502(15); 191.0561(15)	C16H17O8	0.35	*Tran*s-3-Coumaroylquinic Ac.	Perez-Fons 2019[c]; Jaiswal 2011[d]	5.06	*6.62*		
4	6.33	298sh-322	-	367.1025	193.0507(100); 134.0373(10); 173.0456(10)	C17H19O9	-2.6	3-Feruloylquinic ac.	Perez-Fons 2019[c]; Clifford 2003[e]	16.09	*4.79*		
6	8.31	295sh-330	-	295.0459	133.0143(100); 179.0350(20); 115.0037(10)	C13H11O8	-0.1	Caffeoylmalic ac.	Perez-Fons 2019[c]	16.74	*0.26*		
7	8.93	298sh-322	-	367.1024	173.0456(100); 193.0506(20)	C17H19O9	-2.7	4-Feruloylquinic ac.	Perez-Fons 2019[c]; Clifford 2003[e]	nc			
9	11.83	295sh-326	-	309.0616	193.0506(100); 133.0142(25); 134.0373(25)	C14H13O8	0.2	Feruloylmalic ac.	Perez-Fons 2019[c]	16.05	*1.86*		

8	10.61	262; 354	-	625.1407	316.0225(100); 178.09985(5)	C27H29O17	-0.5	Myricetin 3-*O*-rutinoside	Tao 2019[f]	18.26	*0.36*	Flavonols	832.30
10	12.17	258-266sh; 354	-	609.146	300.0277	C27H29O16	-0.2	Quercetin 3-*O*-rutinoside	Tao 2019[f]	556.20	*0.78*		
11	13.4	266-346	-	593.1509	285.0404(100); 284.0326(40)	C27H29O15	-0.3	Kaempferol 3-*O*-rutinoside	Tao 2019[f]; He 2020	240.22	*0.42*		
12	13.8	254-265sh; 354	-	623.1614	315.0510(100); 314.0432(40)	C28H31O16	-0.3	Isorhamnetin derivative (hexoside-pentoside)	Tao 2019[f]	7.48	*5.78*		
13	13.9	354	-	477.103	314.0433(100); 315.0509(20)	C22H21O12	-0.8	Isorhamnetin hexoside	Not described	nc			
14	16.42	258-370	-	301.0354	151.0037(100); 178.9986(80); 121.0295(20)	C15H9O7	0	Quercetin	Standard	3.76	*3.64*		
15	17.53	270-370	-	285.0405	151.0036	C15H9O6	0.1	Kaempferol	Standard	6.39	*4.03*		
16	17.84	270-334	-	537.0823	375.05(100); 417.06(40); 443.0399(30)	C30H17O10	1.2	Robustaflavone	He 2020[g]	58.11	*4.67*	Flavones	88.53
17	18.16	270-334	-	551.0979	375.0499(100); 331.0614(5)	C31H19O10	-0.6	7-*O*-Methylamentoflavone	He 2020[g]	30.42	*4.73*		
1	4.58	278	+	205.0971	188.0706	C11H13O2N2	-0.3	Trypothan	Standard; Nartey 1976[h]	32.92	*1.27*	Other	79.91
5	7.66	274 tail	+	459.0924	139.039(100); 153.0183 (20); 289.0707(10)	C22H19O11	0.5	Epigallocatechin gallate	standard; Perez-Fons 2019[c]	46.99	*3.09*		
												**TOTAL**	**1056.97**
**JUTE MALLOW**													
2	6.09	300sh, 326	-	353.0867	191(100)	C16H17O9	-3.1	5-Caffeoylquinic a	Standard; Azuma 1999[i]	27.48	*1.17*	Hydroxycinnamic acids	163.39
3	6.45	300sh, 326	-	353.0867	173.045(100); 179.0350(80); 191.0561(40)	C16H17O9	-3.2	4-Caffeoylquinic ac.	Yahia 2020[j]	1.57	*7.28*		
4	8.82	295sh, 326	-	367.1024	191.0562(100); 173.0455(20)	C17H19O9	-2.9	5-Feruloylquinic ac.	Guzzeti 2021[k]; Clifford 2003[e]	1.87	*14.13*		
8	13.25	297sh, 326	-	515.119	173.0455(100); 179.0350(90); 353.0868(60); 191.0561(40); 335.0773(30)	C25H23O12	-1	Dicaffeoylquinic ac.	Wagdy 2019[l]	4.06	*6.08*		
9	13.49	297sh, 326	-	515.1191	191.0561(100); 179.0350 (70); 353.0868(50)	C25H23O12	-1.2	3,5-Dicaffeoylquinic ac.	Azuma 1999[i]; Clifford 2006[m]	115.61	*0.56*		
10	14.42	296sh, 326	-	515.1189	173.0455(100); 179.0350(70); 353.0869(70); 191.0561(25)	C25H23O12	-1.1	Dicaffeoylquinic ac.	Wagdy 2019[l]	10.00	*1.68*		

11	15.73	295sh, 326	-	529.1346	191.0561(100); 179.0349(50); 353.0869(20); 367.1025(15); 135.0451(10)	C26H25O12	-1.1	Caffeoylferuloylquinic ac.	Wagdy 2019(l)	2.80	*7.34*		
5	11.98	258-354	-	463.0873	300.0278(100); 301.0355(60)	C21H19O12	-1.8	Quercetin-3-*O*-galactoside	Azuma 1999(i)	24.65	*0.52*		
6	12.3	254-354	-	463.0874	300.0276(100); 301.0354(60)	C21H19O12	-1.7	Quercetin 3-*O*-glucoside	Standard-Azuma 1999(i)	3.76	*2.85*	Flavonols	42.00
7	13.15	255-354	-	549.0982	300.0277(100); 301.0354(50); 505.0981(25); 463.0870(5)	C24H21O15	-0.9	Quercetin 3-(6-malonylhexose)	Azuma 1999(i)	13.58	*2.18*		
1	4.61	278	+	205.0971	188.0708(100)	C11H13O2N2	-0.4	Tryptophan	Standard; Ndamidso 2016(n)	14.94	*0.91*	Other	14.94
												**TOTAL**	**220.33**
**ROSELLE**													
2	3.2	310	-	353.0867	179.0350 (100); 135.0451 (20)	C16H17O9	-2.9	Caffeoylquinic Ac. (isomer)	Beltran-Debon 2010(o); Clifford 2003(e)	9.85	*2.43*		
3	3.98	294sh-326	-	353.0867	191.0561 (100); 179.0350 (80); 135.0451 (20)	C16H17O9	-2.8	3-Caffeoylquinic ac.	Beltran-Debon 2010(o); Clifford 2003(e)	285.48	*0.37*		
5	5.14	306	-	337.093	163.0401(100); 191.0561(40); 119.0502(20)	C16H17O8	0.2	3-Coumaroylquinic ac.	Herranz-Lopez(p) 2012(p); Jaiswal 2011(d)	2.25	*1.86*		
6	5.37	296sh-310	-	337.0931	163.0400(100); 191.0560(10); 119.0502(15)	C16H17O8	0.5	*cis*-3-Coumaroylquinic ac.	Herranz-Lopez(p) 2012(p); Jaiswal 2011(d)	8.57	*0.97*	Hydroxycinnamic acids	365.87
7	5.44	314	-	353.0866	173.0455 (100); 179.0350 (80); 191.0561 (40); 135.0451 (20)	C16H17O9	-3.2	cis-4-Caffeoylquinic ac.	Beltran-Debon 2010(o); Jaiswal 2011(d)	1.13	*8.98*		
8	5.8	300-322	-	179.035	135.0452	C9H7O4	-0.02	Caffeic ac.	Standard; Peng 2011(q)	1.78	*1.12*		
10	6.1	290-326	-	353.0867	191.056	C16H17O9	-2.9	5-Caffeoylquinic ac.	Standard; Beltran-Debon 2010(o)	13.68	*0.36*		
11	6.33	290-322	-	367.1024	193.0506 (100); 134.0373 (10)	C17H19O9	-3	3-Feruloylquinic ac.	Peng 2011; Clifford 2003(e)	3.86	*9.87*		

12	6.43	295sh-326	-	353.0868	173.0455 (100); 179.0350 (80); 191.0561 (40); 135.0451 (20)	C16H17O9	-2.9	4-Caffeoylquinic ac.	Beltran-Debon 2010[(o)]; Jaiswal 2011[(d)]	28.59	*2.45*		
13	6.91	300sh-330	-	369.0452	127.0037(100); 189.0040(50) (ac.e hibiscus); 83.0183(30); 207.0146(10)	C15H13O11	-3	Caffeoylhydroxycitric ac.	Herranz-Lopez 2012[(p)]	4.53	*13.00*		
14	7.42	293sh-314	-	337.0929 (coelué)	191.0561 (100); 173.0455 (15)	C16H17O8	0.17	5-Coumaroylquinic ac.	Herranz-Lopez 2012[(p)] Clifford 2003[(e)]	1.02	*4.98*		
15	7.59	314	-	337.093 (coelué)	173.0455(100); 163.0401(30)	C16H17O8	0.18	4-Coumaroylquinic ac.	Herranz-Lopez 2012[(p)] Clifford 2003[(e)];	0.63	*2.94*		
16	8.65	298sh-326	-	335.0772	161.0244(100); 135.0451(30); 179.0349(10)	C16H15O8	-0.1	5-Caffeoylshikimic ac.	Rodriguez-Medina 2009[(r)]; Clifford 2003[(e)]	3.73	*1.54*		
17	8.89	326	-	367.1022	173.0456 (100); 193,0507 (20)	C17H19O9	-3	4-Feruloylquinic ac.	Peng 2011[(q)]; Clifford 2003[(e)]	0.76	*8.34*		
18	10.89	262-358	-	755.2038	300.0274(100); 201.0710(40); 178.9982	C33H39O20	-0.5	Quercetin derivative (*O*-hexose-didesoxyhexose)	Not described	4.47	*2.10*		
19	11.2	258-354	-	595.1301	300.0276	C26H27O16	-0.55	Quercetin 3-*O*-sambubioside	Rodriguez-Medina 2009[(r)]	5.21	*2.14*		
20	11.88	262-354	-	609.146	300.0275(100); 178.9986(5)	C27H29O16	-0.24	Quercetin derivative (hexose-desoxyhexose)	Not described	10.24	*8.55*		
21	12.18	258-266sh; 303sh-354	-	609.146	300.0276(100); 178.9986(5)	C27H29O16	-0.22	Quercetin 3-*O*-rutinoside	Standard; Rodriguez-Medina 2009[(r)]	297.19	*0.67*		
22	12.29	258-354	-	463.0873	300.0276(100)	C21H19O12	-1.9	Quercetin 3-*O*-glucoside	Rodriguez-Medina 2009[(r)]	87.11	*1.67*	Flavonols	517.67
23	12.78	266-346	-	593.1509	284.0327(100); 285.0404(50)	C27H29O15	-0.46	Kaempferol derivative (hexose-desoxyhexose)	Not described	3.96	*4.07*		
24	13.41	266-346	-	593.1509	284.0326-40); 285.0404(100)	C27H29O15	-0.5	Kaempferol 3-*O*-rutinoside	Rodriguez-Medina 2009[(r)]	92.13	*0.67*		
25	13.54	266-346	-	447.0923	284.0326(100); 285.0403(50)	C21H19O11	-2.2	Kaempferol 3-*O*-glucoside	Standard; not described	11.13	*3.69*		
28	16.43	258-370	-	301.0353	151.0037(100); 178.9986(80); 121.0295(20)	C15H9O7	-0.1	Quercetin	Standard	6.22	*12.95*		

26	13.85	262-272sh; 358	-	609.1246	300.0274(100); 301.0530(100); 463.0868(60)	C30H25O14	-0.1	Quercetin 3-*O*-*p*-coumaroylglucoside	Not described	2.74	*3.79*		
27	16.21	255sh-270-346	-	593.1298	285.0404(100); 284.0326(30); 447.0920(10)	C30H25O13	-0.5	Kaempferol 3-*O*-*p*-coumaroylglucoside	Rodriguez-Medina 2009[r]	1.27	*5.75*	Flavonols cinnamoylglycoside	15.95
29	16.69	266-314-354sh	-	609.125	463.0873(100); 300.0276(70); 301.0353(50)	C30H25O13	-0.1	Quercetin 3-*O*-*p*-coumaroylglucoside (isomer)	Not described	11.94	*26.15*		
1	1.01	298	-	371.0974	201.0709 (100) - 173.0454 (40) - 135.0452 (40)	C16H19O10	-2.7	Unknown		7.64	*7.75*	Other	15.27
4	4.69	282	+	205.0971	188.0706(100)	C11H13O2N2	-0.4	tryptophan	Standard	7.63	*4.24*		
9	5.99	282						Unknown		/	/		
												**TOTAL**	**914.76**
**SPINACH**													
2	5.92	282-tail	-	325.0929	163.04(100); 119.0502(25)	C15H17O8	0.1	coumaric derivative (hexoside?)	Not described	0.54	*0.36*		
3	6.97	310 (HA)	-	295.0459	149.0092(100); 163.0401(70)	C13H11O8	-0.13	cis-coumaroyltartaric ac.	Bergman 2001[s]	1.67	*0.44*	Hydroxycinnamic acids	90.09
4	7.33	314 (HA)	-	295.0459	163.0401(100)	C13H11O8	-0.1	trans-coumaroyltartaric ac.	Bergman 2001[s]	2.47	*0.23*		
7	12.33	314 (HA)	-	337.0566	163.0400(100); 112.988(80); 203.0350(40); 277.0353(15)	C15H13O9	0.2	coumaroyl tartaric acetyl	Bergman 2001[s]	85.41	*1.53*		
8	12.94	258-274sh-318-357sh	-	933.2301	787.1925(100); 330.0380(50); 655.1508(5); 769.1823(10)	C42H45O24	-0.5	Patuletin 3-*O*-(2″-coumaroylglucosyl)(1→6)-[apiosyl(1→2)]-glucoside	Not described	14.11	*2.79*		
10	13.28	254-274sh-334-366sh	-	963.2407	787.1935(100); 330.0382(70); 769.1827(15); 315.0147(5)	C43H47O25	-0.5	Patuletin 3-*O*-(2″-feruloyglucosyl)(1→6)-[apiosyl(1→2)]-glucoside	Ferrerer 1997[t]	27.03	*0.59*		
11	13.86	258-274sh-318-350sh	-	947.2462	801.2092(100); 344.0528(90); 783.1992(30); 329.0304(50)	C43H47O24	-0.1	Spinacetin 3-*O*-(2″-coumaroylglucosyl)(1→6)-[apiosyl(1→2)]-glucoside	Ferrerer 1997[t]	18.66	*3.19*	Flavonols cinnamoylglycoside	122.16
12	14.15	250-274sh-334-370sh	-	977.2565	344.0527 (100); 801.2085(90); 783.1985(40); 329.0300(20)	C44H49O25	-0.3	Spinacetin 3-*O*-(2″-feruloyglucosyl)(1→6)-[apiosyl(1→2)]-glucoside	Not described	40.96	*3.27*		
14	15.29	258-276sh-318-360sh	-	815.2035	345.0606(100); 669.1671(40)	C38H39O20	-0.6	Spinacetin 3-*O*-(2″-coumaroylglucosyl)(1→6)-glucoside	Ferrerer 1997[t]	3.56	*2.71*		

15	15.58	250-274sh-334-370sh	-	845.2141	345.0607(100); 669.1671(30); 330.0382(20)	C39H41O21	-0.5	Spinacetin 3-*O*-(2"-feruloylglucosyl)(1→6)-glucoside	Ferrerer 1997(t)	17.84	*5.28*		
5	11.16	258-350	-	787.1933	330.0382(100); 655.1502(5); 505.0986(5); 373.0547(5); 637.1394(5); 315.0146(5)	C33H39O22	-0.7	patuletin 3-*O*-glucosyl-(1→6)-apiosyl-(1→2)-glucoside	Aritomi 1985(u)	51.38	*1.23*		
6	11.63	258-350	-	655.1517	330.0381(100); 373.0556(5); 315(10)	C28H31O18	0.16	Patuletin 3-*O*-gentiobioside	Aritomi 1985(u)	19.81	*2.71*		
9	13.23	258-271sh-354	-	669.1668	345.0606(100); 344.0528(20); 330.0382(20)	C29H33O18	-0.4	Spinacetin 3-*O*-gentiobioside	Aritomi 1985(u)	33.83	*1.27*	Flavonols	241.62
13	14.94	270-342	-	521.0931	345.0606(100); 330.0381(70); 315.0145(5)	C23H21O14	-0.5	Axillarin 4'-*O*-glucuronide	Aritomi 1985(u)	102.35	*1.60*		
16	16.07	254-270-342	-	535.1088	344.0528(100); 359.0763-80); 329.0302(15)	C24H23O14	-0.9	Jaceindine 4'-*O*-glucuronide	Aritomi 1984(v)	34.26	*0.82*		
17	17.17	253-278-342	-	519.0775	343.0446(100); 328.0225(60); 113.0244(5)	C23H19O14	-0.5	5,3',4'-trihydroxy-3-methoxy-6:7-methyldioxyflavone-(4'-glucuronide)	Aritomi 1984(v), compound 1	43.69	*2.04*		
18	17.32	255sh-278-334	-	503.0822	327.0510(100); 312.0275(80); 113.0244 (70)	C23H19O13	-1.6	5,4'-dihydroxy-3-methoxy-6:7-methyldioxyflavone-(4'-glucuronide)	Not described	1.63	*3.11*	Flavones	56.67
19	17.41	254-278-342	-	533.0934	357.0601(100); 342.0367(100); 327.0146(5); 113.0244(40)	C24H21O14	-0.5	5,4'-dihydroxy-3,3'-dimethoxy-6:7-methyldioxyflavone-(4'-glucuronide)	Aritomi 1984(v), composé 7	14.35	*3.69*		
1	4.6	278	+	205.0971	188.0707(100)	C11H13O2N2	-0.2	tryptophan	Standard	6.86	*1.73*	Other	6.86
												**TOTAL**	**520.39**

Values are expressed as mean ± SD (n = 3). RSD: Relative Standard Deviation (%) ; Conc.: Concentration ; Ac.: Acid

(a) [84]; (b) [100]; (c) [101]; (d) [72]; (e) [70]; (f) [17]; (g) [102]; (h) [103] ; (i) [104] ; (j) [105] ; (k) [106]; (l) [107]; (m) [71]; (n) [108] ; (o) [109] ; (p) [110]; (q) [111]; (r) [73] ; (s) [112]; (t) [113]; (u) [114] ; (v) [115]

References

17. Tao, H.; Cui, B.; Zhang, H.; Bekhit, A.E.-D.; Lu, F. Identification and Characterization of Flavonoids Compounds in Cassava Leaves (Manihot Esculenta Crantz) by HPLC/FTICR-MS. *Int. J. Food Prop.* **2019**, *22*, 1134–1145, doi:10.1080/10942912.2019.1626879.
70. Clifford, M.N.; Johnston, K.L.; Knight, S.; Kuhnert, N. Hierarchical Scheme for LC-MSn Identification of Chlorogenic Acids. *J. Agric. Food Chem.* **2003**, *51*, 2900–2911.
71. Clifford, M.N.; Knight, S.; Surucu, B.; Kuhnert, N. Characterization by LC-MSn of Four New Classes of Chlorogenic Acids in Green Coffee Beans: Dimethoxycinnamoylquinic Acids, Diferuloylquinic Acids, Caffeoyl-Dimethoxycinnamoylquinic Acids, and Feruloyl-Dimethoxycinnamoylquinic Acids. *J. Agric. Food Chem.* **2006**, *54*, 1957–1969.
72. Jaiswal, R.; Deshpande, S.; Kuhnert, N. Profiling the Chlorogenic Acids of Rudbeckia Hirta, Helianthus Tuberosus, Carlina Acaulis and Symphyotrichum Novae-Angliae Leaves by LC-MSn. *Phytochem. Anal.* **2011**, *22*, 432–441.
73. Rodríguez-Medina, I.C.; Beltrán-Debón, R.; Molina, V.M.; Alonso-Villaverde, C.; Joven, J.; Menéndez, J.A.; Segura-Carretero, A.; Fernández-Gutiérrez, A. Direct Characterization of Aqueous Extract of Hibiscus Sabdariffa Using HPLC with Diode Array Detection Coupled to ESI and Ion Trap MS. *J. Sep. Sci.* **2009**, *32*, 3441–3448, doi:10.1002/jssc.200900298.
84. Neugart, S.; Baldermann, S.; Ngwene, B.; Wesonga, J.; Schreiner, M. Indigenous Leafy Vegetables of Eastern Africa — A Source of Extraordinary Secondary Plant Metabolites. *Food Res. Int.* **2017**, *100*, 411–422, doi:10.1016/j.foodres.2017.02.014.
100. Klimczak, I.; Małecka, M.; Pachołek, B. Antioxidant Activity of Ethanolic Extracts of Amaranth Seeds. *Food/Nahrung* **2002**, *46*, 184–186.
101. Perez-Fons, L.; Bohorquez-Chaux, A.; Irigoyen, M.L.; Garceau, D.C.; Morreel, K.; Boerjan, W.; Walling, L.L.; Becerra Lopez-Lavalle, L.A.; Fraser, P.D. A Metabolomics Characterisation of Natural Variation in the Resistance of Cassava to Whitefly. *BMC Plant Biol.* **2019**, *19*, 518.
102. He, C.-W.; Wei, J.-H.; Zeng, L.-Y.; Deng, J.-G. Triterpenoids and Flavonoids from Cassava Leaves. *Chem. Nat. Compd.* **2020**, *56*, 331–333.
103. Nartey, F.; Møller, B.L. Amino Acid Profiles of Cassava Seeds (Manihot Esculenta). *Econ. Bot.* **1976**, *30*, 419–423.
104. Azuma, K.; Nakayama, M.; Koshioka, M.; Ippoushi, K.; Yamaguchi, Y.; Kohata, K.; Yamauchi, Y.; Ito, H.; Higashio, H. Phenolic Antioxidants from the Leaves of Corchorus Olitorius L. *J. Agric. Food Chem.* **1999**, *47*, 3963–3966, doi:10.1021/jf990347p.
105. Yahia, Y.; Bagues, M.; Tlahig, S.; Lazreg, M.; Loumerem, M.; Nagaz, K. Metabolites Secondaires et Activités Biologiques de Corètes (Corchorus Olitorius L.) Cultivées Dans Le Sud Tunisien. *Revue R.A.* **2021**.
106. Guzzetti, L.; Panzeri, D.; Ulaszewska, M.; Sacco, G.; Forcella, M.; Fusi, P.; Tommasi, N.; Fiorini, A.; Campone, L.; Labra, M. Assessment of Dietary Bioactive Phenolic Compounds and Agricultural Sustainability of an African Leafy Vegetable Corchorus Olitorius L. *Front. Nutr.* **2021**, *8*, 667812, doi:10.3389/fnut.2021.667812.
107. Wagdy, R.; Abdelkader, R.M.; El-Khatib, A.H.; Linscheid, M.W.; Hamdi, N.; Handoussa, H. Neuromodulatory Activity of Dietary Phenolics Derived from Corchorus Olitorius L. *J. Food Sci.* **2019**, *84*, 1012–1022, doi:https://doi.org/10.1111/1750-3841.14587.
108. Ndamitso, M.M.; M.Jagaba, M.M.; Musah, J.I.; Mann, A.; E.B., M.; A., U. Chemical Composition, Antinutrient Contents and Functional Properties of Corchorus Olitorius and Melochia Corchorifolia. *Trends Food Sci. Technol.* **2016**, *1*, 96–100.
109. Beltrán-Debón, R.; Alonso-Villaverde, C.; Aragonès, G.; Rodríguez-Medina, I.; Rull, A.; Micol, V.; Segura-Carretero, A.; Fernández-Gutiérrez, A.; Camps, J.; Joven, J. The Aqueous Extract of Hibiscus Sabdariffa Calices Modulates the Production of Monocyte Chemoattractant Protein-1 in Humans. *Phytomedicine* **2010**, *17*, 186–191.
110. Herranz-López, M.; Fernández-Arroyo, S.; Pérez-Sanchez, A.; Barrajón-Catalán, E.; Beltrán-Debón, R.; Menéndez, J.A.; Alonso-Villaverde, C.; Segura-Carretero, A.; Joven, J.; Micol, V. Synergism of Plant-Derived Polyphenols in Adipogenesis: Perspectives and Implications. *Phytomedicine* **2012**, *19*, 253–261.

111. Peng, C.-H.; Chyau, C.-C.; Chan, K.-C.; Chan, T.-H.; Wang, C.-J.; Huang, C.-N. Hibiscus Sabdariffa Polyphenolic Extract Inhibits Hyperglycemia, Hyperlipidemia, and Glycation-Oxidative Stress While Improving Insulin Resistance. *J. Agric. Food Chem.* **2011**, *59*, 9901–9909.
112. Bergman, M.; Varshavsky, L.; Gottlieb, H.E.; Grossman, S. The Antioxidant Activity of Aqueous Spinach Extract: Chemical Identification of Active Fractions. *Phytochem* **2001**, *58*, 143–152.
113. Ferreres, F.; Castañer, M.; Tomás-Barberán, F.A. Acylated Flavonol Glycosides from Spinach Leaves (Spinacia Oleracea). *Phytochem.* **1997,** *45*, 1701–1705.
114. Aritomi, M.; Komori, T.; Kawasaki, T. Flavonol Glycosides in Leaves of Spinacia Oleracea. *Phytochem.* **1985**, *25*, 231–234.
115. Aritomi, M.; Kawasaki, T. Three Highly Oxygenated Flavone Glucuronides in Leaves of Spinacia Oleracea. *Phytochem.* **1984**, *23*, 2043–2047.
